# Improved Resolution and Reduced Clutter in Ultra-Wideband Microwave Imaging Using Cross-Correlated Back Projection: Experimental and Numerical Results

**DOI:** 10.1155/2010/781095

**Published:** 2011-01-23

**Authors:** S. Jacobsen, Y. Birkelund

**Affiliations:** Department of Physics and Technology, Faculty of Science & Technology, University of Tromsø, 9037 Tromsø, Norway

## Abstract

Microwave breast cancer detection is based on the dielectric contrast between healthy and malignant tissue. This radar-based imaging method involves illumination of the breast with an ultra-wideband pulse. Detection of tumors within the breast is achieved by some selected focusing technique. Image formation algorithms are tailored to enhance tumor responses and reduce early-time and late-time clutter associated with skin reflections and heterogeneity of breast tissue. In this contribution, we evaluate the performance of the so-called cross-correlated back projection imaging scheme by using a scanning system in phantom experiments. Supplementary numerical modeling based on commercial software is also presented. The phantom is synthetically scanned with a broadband elliptical antenna in a mono-static configuration. The respective signals are pre-processed by a data-adaptive RLS algorithm in order to remove artifacts caused by antenna reverberations and signal clutter. Successful detection of a 7 mm diameter cylindrical tumor immersed in a low permittivity medium was achieved in all cases. Selecting the widely used delay-and-sum (DAS) beamforming algorithm as a benchmark, we show that correlation based imaging methods improve the signal-to-clutter ratio by at least 10 dB and improves spatial resolution through a reduction of the imaged peak full-width half maximum (FWHM) of about 40–50%.

## 1. Introduction

As early-stage tumor detection is a prognostic key factor when curing breast cancer [1], the prevailing driving force in emerging screening technologies is the ability to identify increasingly smaller tumors during the initial nonpalpable phase of the disease. Conventional imaging modalities like X-ray mammography, computed tomography (CT), magnetic resonance imaging (MRI), and positron emission tomography (PET) scanning all provide critical information for clinicians during diagnosis and status evaluation of breast cancer. However, the latter three methods are associated with relatively large running costs. Thus, mammography remains the most common technique for breast cancer screening. Although the modality provides high-resolution images, it is based on ionizing radiation and requires uncomfortable or painful compression of the breast. Additionally, the method is of limited value for younger woman with radiographically dense breast tissue. The inherent limitations of X-ray mammography are widely recognized in terms of significant false-positive [2] and false-negative [3] diagnostic outcomes from mammograms.

Variable dielectric properties (electrical permittivity and conductivity) of breast tissue composition offer an alternative contrast mechanism within a substantial range of the electromagnetic spectrum. Motivated by the much higher dielectric contrast between normal and malignant tissue in both the radio-frequency (~1 MHz) and microwave (1–11 GHz) regions, complementary technologies are under investigation for early-stage breast cancer detection. Three different approaches to active imaging, including electrical and microwave impedance tomography [4], backscatter methods [5], and microwave-induced acoustic imaging [6] have been proposed in the literature. In addition, passive infrared thermography [7] and microwave radiometry [8], that utilize the thermal contrast between cancerous and normal tissues, are also under investigation. Overall, electromagnetic (EM) breast imaging provides a low-cost and safe alternative, with the potential to provide higher specificity than other conventional imaging approaches.

The inverse scattering approach (originally adapted from ground penetrating radar [9]) involves illumination of the breast with an ultra-wideband (UWB) pulse from either an array of applicators or mechanically scanned antenna(s). Based on sophisticated, yet robust, focusing techniques, confocal microwave imaging (CMI) seeks to determine the location of strong scatterers within the breast [10]. In contrast to proposed tomographic systems, radar-based systems operate at higher frequencies (up to about 10 GHz) with larger bandwidths (up to about 9 GHz). Compared to tomographic techniques, radar-based imaging requires relatively simple signal processing algorithms for image-formation.

Although encouraging experimental phantom results have been published in the literature, an extremely low signal-to-clutter ratio (SCR) of the backscattered tumor signature can be anticipated in clinical scenarios, owing to competing scatter information from other dominant sources like skin, antenna, and tissue heterogeneities. Furthermore, microwave imaging methods do not offer spatial resolution better than a few millimeters as a consequence of limited bandwidth and pulse distortion due to tissue and spherical losses. Thus signal clutter and spatial resolution are among the most important performance factors that need to be addressed in further development of this method. In this paper, we demonstrate through phantom experiments how both the SCR and spatial resolution can be improved by utilizing existing similarities (pulse decorrelation length and pulse shape), in the tumor response across adjacent channels, through implementation of so-called cross-correlated back projection methods.

The following section presents our scheme for beamformer design including imaging algorithms, a skin-breast artifact removal algorithm, and a 2D image formation procedure.  Section 3 describes the experimental setup. In Section 4, the efficiency of the triple-correlation back projection scheme is demonstrated both experimentally and numerically.  Section 5 discusses the obtained results and Section 6 draws conclusions from the work. 

## 2. Methodology

### 2.1. Signal Processing

#### 2.1.1. Imaging Algorithms

Assume that the basic imaging setup consists of antennas *A*_*i*_, *i* = 1,…, *Q* stimulating a medium under investigation with a UWB pulse. Depending on the data acquisition system and concept to be realized, monostatic [11], bistatic [12], and multistatic [13] approaches have been implemented numerically and/or experimentally in medical UWB radar imaging. Theoretically, the imaging methods map a point object response from the respective signals as arcs (parts of an ellipse) in the migrated image. All scattered information is superimposed such that the different arcs in principle only intercept at the point scatterer location. Image formation using the well known delay-and-sum (DAS) [14] algorithm can be stated

(1) $$\begin{array}{c} F_{\text{DAS}}\left(\underline{r}\right)={\left(\overset{Q}{\underset{i=1}{\sum }}w_{i}\left(\underline{r}\right)\int_{-T/2}^{T/2}S_{i}\left[t-\varsigma_{i}\left({\underline{r}}_{0}\right)+\tau_{i}\left(\underline{r}\right)\right]dt\right)}^{2}, \end{array}$$

where *T* is the length of the integration window (related to the system bandwidth), $\underline{r}$ is a spatial image variable, *w*_*i*_ is a location dependent weighting function, *S*_*i*_ is the radar signal (for illustration purposes assumed to be a time-shifted replica of the transmitted pulse), *ς*_*i*_ is the signal time-delay relative to a point scatterer (at position ${\underline{r}}_{0}$) and antenna #*i*, and *τ*_*i*_ is the time-shift used for each focal point in the image. *τ*_*i*_ is related to the round-trip propagation distance through *τ*_*i*_ = (*r*_*i*_^*T*_*X*_^ + *r*_*i*_^*R*_*X*_^)/*v*_*m*_ where *r*_*i*_^*T*_*X*_^ and *r*_*i*_^*R*_*X*_^ are the transmitter-to-point scatterer and point scatterer-to-receiver distances, respectively and *v*_*m*_ is the medium speed.

The signals sum completely only when $\varsigma ({\underline{r}}_{0})=\tau_{i}(\underline{r})$ if *S*_*i*_ can be approximated by a Dirac delta-function. However, due to limited spatial resolution, the summation also causes some artifacts that are distributed throughout the image. The summation will in practice be partly coherent in the vicinity of the peak producing resolution degradation and side lobes. Furthermore, away from the mapped peak, clutter will also appear as a consequence of contributions from each individual antenna signal.

These properties can be illustrated by the following simplified analytical 2D model. Assume two omnidirectional antennas monostatically operated and located at positions (*x*_*a*_, *y*_*a*_) = (±*D*/2, 0),0), together with a point scatterer located at (*x*_*s*_, *y*_*s*_) = (0, *y*_0_). Further, assume that the received signal can be approximated by a demodulated Gaussian pulse as *S*(*t*) = exp (−*t* ^2^/*σ*_*t*_^2^), where *σ*_*t*_ is the characteristic pulse-width in the time domain. Substitution of these parameters into (1) results in the far-field approximation:

(2) $$\begin{array}{c} F_{\text{DAS}}\left(\xi ,y=y_{0}\right)\approx \exp\;\left(-\frac{2\left(\xi^{2}+\xi^{4}\right)}{\rho^{2}{ɛ}^{2}}\right){\cosh\;}^{2}\left(\frac{\xi^{2}}{\rho^{2}{ɛ}^{2}}\right), \end{array}$$

where the following dimensionless parameters have been defined: *ξ* = *x*/*D* (normalized cross-range), *ρ* = *σ*_*t*_ *v*/*D* = *σ*_*x*_/*D* (normalized spatial pulse width), and *ɛ* = *y*_0_/*D* (normalized range distance) assuming *y*_0_ ≫ *D*.

**Figure 1:**
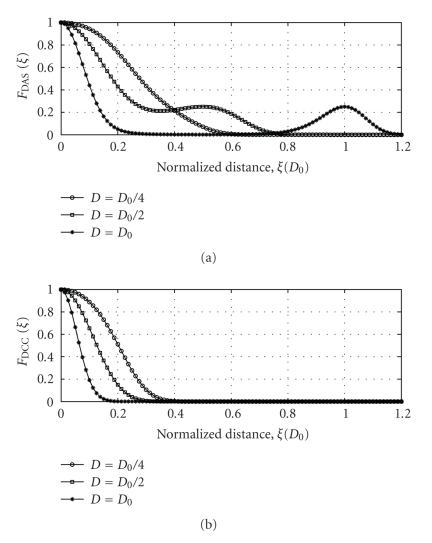
1D profile response versus lateral normalized distance *ξ*. (a) Delay-and-sum algorithm (2), (b) Dual cross-correlated back projection algorithm (4). Normalized parameter values. *ρ* = 0.025 and *ɛ* = 6.25.25 for *D* = *D*_0_.

Interpretation of (2) reveals that the exponential term is monotonously decreasing with *ξ* whereas the hyperbolic cosine function increases with *ξ*. The latter term will contribute to clutter-like side lobes along the cross-range direction away from the main lobe as illustrated in Figure 1 (upper panel), where the 1D response in the transversal (cross-range) direction is plotted as a function of distance *D* between the antennas. As expected, the main lobe narrows with increasing *D*; but at the expense of appearing side lobes.

Foo and Kashyap [15] addressed this problem and suggested a method to suppress side lobes and improve image resolution. Their solution is joint migration of pairwise signals, one gathered by a receiver *A*_*i*_ and the other by an auxiliary reference receiver *A*_*i*_^*R*^. Summing several products of such pairs leads to the dual cross-correlated (DCC) back projection algorithm, which can be stated

(3) $$\begin{array}{cc} F_{\text{DCC}}\left(\underline{r}\right) & =(\overset{Q}{\underset{i=1}{\sum }}w_{i}\left(\underline{r}\right)\int_{-T/2}^{T/2}S_{i}\left[t-\varsigma_{i}\left({\underline{r}}_{0}\right)+\tau_{i}\left(\underline{r}\right)\right]\\ & \;\;\;{\cdot {\tilde{S}}_{i}^{R}\left[t-\varsigma_{i}^{R}\left({\underline{r}}_{0}\right)+\tau_{i}^{R}\left(\underline{r}\right)\right]dt)}^{2}. \end{array}$$

In (3), the reference signal ${\tilde{S}}_{i}^{R}$ is normalized within the integration window *T* to produce the same power level as the rectangular window in (1).

The above 2D analytical scenario can also be used to derive characteristics of this imaging scheme. The product of the left and right antenna signals, with proper numerical parameter values substituted into (3) yields

(4) $$\begin{array}{c} F_{\text{DCC}}\left(\xi ,y=y_{0}\right)\approx \exp\;\left(-\frac{4\left(\xi^{2}+\xi^{4}\right)}{\rho^{2}{ɛ}^{2}}\right) \end{array}.$$

By comparing (2) and (4) we observe that the main difference is in the hyperbolic cosine term (which broadens the response) in addition to a faster decaying exponential term in (4). Equation (4) is plotted in Figure 1 (lower panel) for different antenna distances. The improved performance of the correlation method compared to the delay-and-sum method is corroborated through significantly narrower mainlobes (better resolution) and lack of sidelobes (reduced clutter) in the former case.

The philosophy behind the cross-correlated back projection algorithm is to combine two ellipses that intersect in the point scatterer location and to utilize the short decorrelation length of the pulses. Zetik et al. [16] suggested a modification of this scheme by exploiting a second reference antenna to form a sum of triple products as follows:

(5) $$\begin{array}{cc} F_{\text{TCC}}\left(\underline{r}\right) & =(\overset{Q}{\underset{i=1}{\sum }}w_{i}\left(\underline{r}\right)\int_{-T/2}^{T/2}S_{i}\left[t-\varsigma_{i}\left({\underline{r}}_{0}\right)+\tau_{i}\left(\underline{r}\right)\right]\\ & \;\;\;\cdot {\tilde{S}}_{i}^{R_{1}}\left[t-\varsigma_{i}^{R_{1}}\left({\underline{r}}_{0}\right)+\tau_{i}^{R_{1}}\left(\underline{r}\right)\right]\\ & \;\;\;{\cdot {\tilde{S}}_{i}^{R_{2}}\left[t-\varsigma_{i}^{R_{2}}\left({\underline{r}}_{0}\right)+\tau_{i}^{R_{2}}\left(\underline{r}\right)\right]dt)}^{2}, \end{array}$$

where the superscripts *R*_1_ and *R*_2_ refer to the auxiliary reference receiver pair. Being a generalization of (3), this approach is denoted triple cross-correlated (TCC) back projection beamforming.

**Figure 2:**
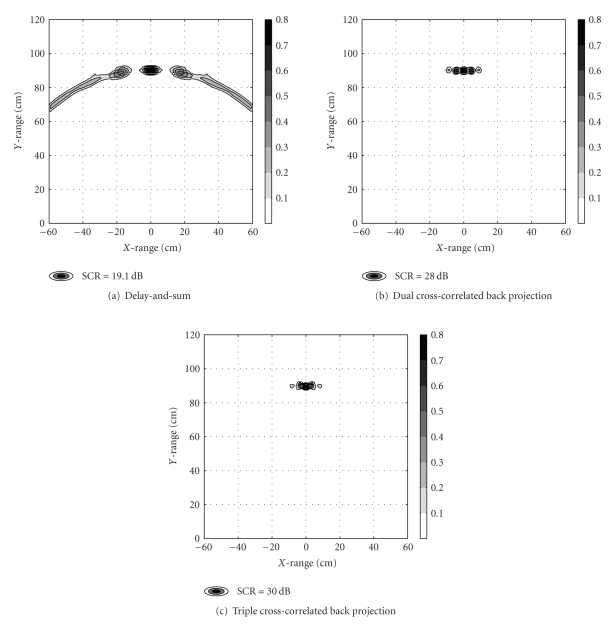
Grey-scale images of spatial power deposition in numerical test scenario.

In what follows, the performance of the three imaging algorithms will be preliminary evaluated in a second test scenario using numerical data generated by the commercial EM solver CST MWS (http://www.cst.com/). Assume a monostatic arrangement of five identical antenna elements in a linear array configuration along the *x*-axis. Horn antennas were used with aperture dimensions of 25.5 × 12.5 cm^2^ and each antenna was fed by an S-band waveguide (7.2 × 3.4 cm^2^) which again was excited by an impedance-matched port. Only the TE_10_-mode, with a lower cut-off frequency of 2.0 GHz, was considered in the simulations. The lateral distance between the center points of two adjacent antennas was 13 cm. To obtain a point-spread response, a tiny sphere (Ø5 mm) of PEC material was located in the immersion medium (air) at the symmetry line (*y*-axis) 90 cm away from the center element aperture. Each port transmitted a UWB (modulated Gaussian) stimulation pulse centered at 6 GHz with a bandwidth from 1 to 11 GHz (relative to 10% of the spectral maximum). To remove any clutter from the early-time response, the same runs were conducted with the scatterer removed. Residual signals for image formation were obtained by subtracting the scatterer-free runs from the original data set. Figure 2 depicts the 2D image generated by the three methods. Visual inspection confirms the inherent property of nonnegligible side lobes associated with the delay-and-sum scheme. In order to evaluate this phenomenon, the overall performance was quantified by the signal-to-clutter ratio (SCR), presently defined as SCR = *P*_peak_/〈*P*〉, where *P*_peak_ is the peak maximum power value and 〈*P*〉 is the average power level outside the −3 dB peak area. Furthermore, the analytical expressions above indicate that the correlation methods also improve the spatial resolution. In order to evaluate the tumor response and extent, the full width half maximum (FWHM) was derived along both axes.  Table 1 summarizes the calculated performance indices. With SCR-values of 9–11 dB higher than the delay-and-sum algorithm, the two cross-correlated methods perform significantly better. Also, FWHM_*x*_ (cross-range peak width) is recorded as less than half the peak width for the correlation methods when comparing the two approaches.

Another important aspect of the correlation methods is how to form the signal products. That is, what reference antenna(s) to use together with antenna *A*_*i*_. Two configurations have been implemented: (1) Sequential configuration: *A*_*i*_ combined with *A*_*i*+1_ (dual cross-correlation) or *A*_*i*_ combined with *A*_*i*+1_ and *A*_*i*−1_ (triple cross-correlation); (2) Interlaced configuration: *A*_*i*_ combined with *A*_*i*+*n*_, *n* > 1 (dual cross-correlation) or *A*_*i*_ combined with *A*_*i*+*n*_ and *A*_*i*−*n*_ (triple cross-correlation). The rationale for the interlaced implementation can be seen in Figure 1, which shows that the resolution improves with the antenna distance *D*. Hence, combinations of adjacent antennas are not expected to produce optimum results. The numerical example, in which *n* = 2 is used for the interlaced configuration, confirms this property (data not shown) through a 1-2 dB increase in SCR. Interlaced implementation also showed superior performance for experimental data (see Results and Discussion sections).

#### 2.1.2. Removal of Early-Time Response

A major challenge in radar-based imaging of the breast is to reduce the large reflection from the skin-normal tissue interface, as this signal can be several orders of magnitude larger than the tumor response. The reflected pulse is typically dominated by clutter arising from the incident pulse, reflections from the skin layer, and residual antenna reverberations. However, skin reflection is expected to be the dominant component of the backscattered pulse if the antenna is well matched to the immersion medium across the operating bandwidth.

The recursive least-square (RLS) algorithm has been used extensively within fields like adaptive system identification, filtering, and prediction. This algorithm has also been applied with success for effective skin response subtraction in UWB breast phantom experiments [17]. Principally, it differs from the Microwave Imaging via Space Time (MIST) [18] skin subtraction and channel averaging [19] approaches in that the filter weights are updated dynamically throughout the filtering process.

In our experience, using a large number of antennas, the degrees-of-freedom (filter weights) can be reduced in the RLS algorithm without degrading the performance of the filter. Presently, a modified RLS algorithm based on clustered signals is implemented. In order to make the paper self-contained, we outline the modifications of the RLS algorithm.

Assume a data matrix consisting of *Q* × *N* samples, where *Q* and *N* are the number of channels (antennas) and samples pr channel, respectively. To account for nonstationarity in the early-time response, the signals are grouped into *M* = *Q*/*J* subsets from *J* neighboring antennas in which the mutual signal correlation is high. Within each subset, a template signal ${\underline{\tilde{u}}}_{i},i=1,\ldots ,M$, is formed by ensemble averaging across the subset. Now, define ${\underline{u}}_{i}$ as the signal response from each antenna. Without loss of generality, assume that the early-time response is to be removed from ${\underline{u}}_{1}\equiv \underline{d}$ (1 × *N* vector) where a desired signal $\underline{d}$ is defined. The remaining signals are clustered (as outlined above) and used to form the *M* × *N* matrix $\tilde{u}={\left[{\underline{\tilde{u}}}_{1},{\underline{\tilde{u}}}_{2},\ldots ,{\underline{\tilde{u}}}_{M}\right]}^{T}$. Similarly, a *M* × 1 weight vector is defined as $\underline{w}(n)={\left[w_{1}(n),w_{2}(n),\ldots ,w_{M}(n)\right]}^{T}$. The approximation of the desired signal at time instant *n* can be stated as:

(6) $$\begin{array}{c} \hat{d}\left(n\right)={\underline{w}}^{T}\left(n\right)\underline{\tilde{u}}\left(n\right), \end{array}$$

together with the weighted least square error:

(7) $$\begin{array}{c} C\left(n\right)=\overset{n}{\underset{i=1}{\sum }}\lambda^{n-i}{\left|d\left(i\right)-\hat{d}\left(i\right)\right|}^{2}, \end{array}$$

where *λ* ∈ (0,1) is the forgetting factor that limits the number of input samples *C*(*n*) is based on. The smaller *λ* is, the smaller contribution from previous samples. *λ* = 1 is referred to as the growing window algorithm.

Following [17], we end up with the basic equation for the filter coefficients:

(8) $$\begin{array}{c} \underline{w}\left(n\right)=R^{-1}\left(n\right)\underline{z}\left(n\right), \end{array}$$

where

(9) $$\begin{array}{c} R\left(n\right)=\overset{n}{\underset{i=1}{\sum }}\lambda^{n-i}\tilde{u}\left(i\right)\tilde{u}{\left(i\right)}^{T},\\ \underline{z}\left(n\right)=\overset{n}{\underset{i=1}{\sum }}\lambda^{n-i}\tilde{u}\left(i\right)d^{T}\left(i\right). \end{array}$$

Solving for $\underline{w}(n)$ in (8) requires a matrix inversion of **R**. Since **R** tends to be ill-conditioned, a Woodbury matrix identity is used to invert this matrix recursively.

The performance of the RLS algorithm in terms of removing the skin response and not distorting the tumor response was tested for different values of the clustering parameter *M*. In the numerical computations, the early-time responses are very similar (marginally different due to local grid variations) for all channels. Hence, excellent results were obtained by using *Q* = *J* reducing the problem to finding only one filter weight (*M* = 1). This approach is similar to subtracting the channel ensemble average from each signal, but applying time-variable weighting. For the experimental data, marked variations in the early-time response were observed as a consequence of instrument drift during data acquisition. Clustering of eight neighboring antennas into three subsets (*M* = 3) provided sufficient flexibility in the filter to remove the skin response with minimum distortion of the tumor response. As *M* was further increased, the RLS filter showed a tendency of also removing the tumor response from the data.

#### 2.1.3. Image Formation

Time-domain backscattered waveforms were synthesized from measured *S*_11_-parameter frequency domain scans. Before applying the focusing algorithms, the following preprocessing steps need to be performed: (i) a calibration signal obtained by loading the antenna with the immersion medium only was subtracted from all received pulses, (ii) all channels were range-gated to a time interval that falls within the dimensional extent of the phantom, (iii) the skin response was removed from each channel by applying the RLS algorithm outlined above, and (iv) equalization was conducted for each channel to compensate for radial spread of the wavefront (antenna angular radiation patterns and tissue losses were neglected).

The time delay *τ*_*i*_ for a given channel is calculated based on the antenna-to-focal point distance $\underline{r}$. Implicitly, this means that the phase center of the elliptical antenna is assumed to be constant irrespective of observation angle and frequency. This assumption is only viable within the main lobe of the antenna radiation pattern. During processing, the focal point was moved in the center plane (*z* = 0), resulting in a 2D mapping of scattered energy.

## 3. Experimental and Numerical Setup

**Figure 3:**
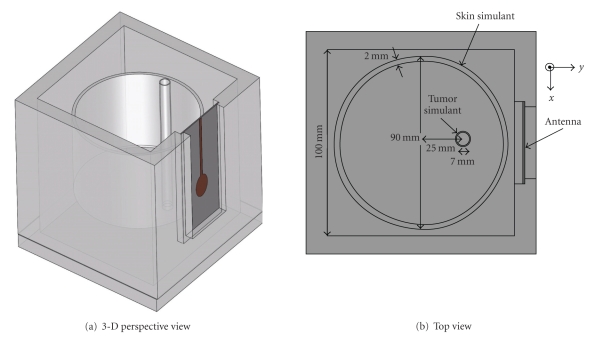
Schematics of experimental/numerical setup.

The experimental setup shown in Figure 3 emulated a system configuration where a patient is lying in a prone position with a synthetic cylindrical antenna array surrounding the breast. The breast phantom consists of a plexiglass box (10.5 × 10.5 × 12 cm^3^) filled with a liquid that mimics the normal breast tissue (soybean oil with dielectric properties *ϵ*_*r*_ = 2.6 and *σ* = 0.05 S/m at 6 GHz), a thin-walled (2 mm) Pyrex glass cylinder (*ϵ*_*r*_ = 5.0 and loss tangent tan*δ* = 0.054) representing the skin, and a thin Pyrex glass cylinder (1 mm wall thickness and 7 mm in diameter) filled with a Tween-60 mixture (*ϵ*_*r*_ = 10 and *σ* = 0.3 S/m at 6 GHz) as the tumor simulant. Soybean oil was chosen as the normal tissue material because of its availability, nontoxicity, and dielectric properties being similar to very low water content fatty tissue. These substances give a contrast of 1 : 2 between skin and normal tissue simulants, which dependent on the fibroglandular-to-adipose distribution, is close to the upper bound of measured * ex vivo* data [20]. The normal-to-malignant tissue contrast is 1 : 4 and thus falls within the upper range for adipose-dominated tissue [20]. The skin simulant (Pyrex glass) has a dielectric constant in-between the normal and malignant tissue simulants. This system is designed for preliminary method verification with materials that have similar contrasts in electrical properties to those expected in the breast. However, since tissue loss is anticipated to limit the performance, spatial resolution and sensitivity are expected to be degraded in more realistic setups due to the impact on system bandwidth and measured tumor response. 

The antenna was immersed in a matching medium which is equivalent to the normal tissue phantom liquid. During data collection, the cylindrical tumor simulant was rotated in 24 positions (15 degrees increment) within the box to synthesize the antenna scan around the breast model. The antenna was connected to an HP8719D (50 MHz–13.5 GHz) vector network analyzer (VNA) to transmit and receive microwave signals. The VNA performed a linear 401 point frequency sweep from 1–12 GHz. From the *S*_11_-parameter, the time domain backscattered pulses were derived by inverse FFT (multiplied by a Gaussian window to limit pulse ringing and to comply with the numerical model). An example of triplets using 24 antennas with interlaced configuration in (5) is [A1  A4  A7], [A2  A5  A8], [A3  A6  A9], and so forth. The sequential configuration applies adjacent antenna subset combinations during beamforming.

**Figure 4:**
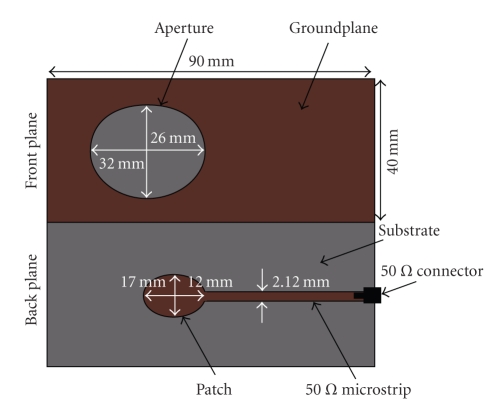
Antenna layout of UWB elliptical antenna used in the microwave imaging system.

The UWB antenna used for transceiving microwave energy is a modified version of a low-profile, single-ended, planar, elliptical antenna. A generic form for use within UWB communication was proposed in [21]. However, the original design was presently customized by moving the radiating patch from the front aperture plane to the back plane and using microstrip feed instead of direct SMA connection at the input terminals. This design modification made the production process much more repeatable without degrading the bandwidth performance. The planar elliptical antenna used for UWB stimulation is shown in Figure 4. The elliptical patch at the back plane was designed with major and minor axes of *a*_*p*_ = 17 mm and of *b*_*p*_ = 12 mm (ellipticity ratio 1.4), respectively. Further, the elliptical aperture in the front plane was designed with major and minor axes of *a*_*a*_ = 32 mm and *b*_*a*_ = 26 mm (ellipticity ratio 1.23), respectively. The dimensional design parameters were obtained via a systematic numerical investigation of ellipticity combinations that gave a return loss better than −10 dB throughout the UWB spectrum. An antenna impedance as insensitive as possible to load variations was also emphasized in the antenna design. Teflon (PTFE) laminate (https://www1.elfa.se/) with substrate thickness *h* = 0.8 mm, 35 *μ* C, relative permittivity *ϵ*_*l*_ = 2.75, and loss tangent 0.0030 (at 10 GHz) was used for the realization. As discussed above, the antenna was fed in the back plane by a 50 Ω microstrip transmission line connected to the VNA via an SMA connector (see Figure 4). 

**Figure 5:**
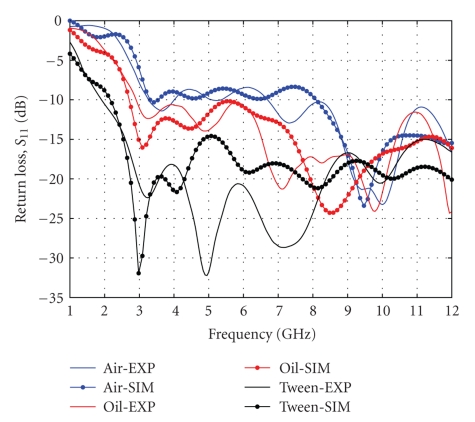
Return loss of elliptical antenna for different loads.

Measured return loss of the elliptical antenna is shown in Figure 5 for different loads. The antenna was (as expected) electrically smallest when loaded by air. As the dielectric constant of the load increases, the lower cut-off frequency decreases. In the experiment, soybean oil was used giving an effective bandwidth range of 2–12 GHz. In addition, good overall agreement between measured and simulated return loss was observed where existing discrepancies were mostly due to small connector mismatches.

## 4. Results 

**Figure 6:**
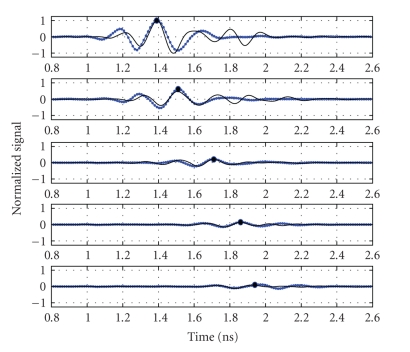
Subset of backscattered signals after early-time response removal (from top to bottom) for antenna #7 (closest to the tumor), #10, #13, #16, #19 (farthest from the tumor). Solid line: experiment, (•): simulations. Black bullet marks expected signal time-of-arrival for each channel.

This section presents experimental and supporting numerical results of phantom tumor detection obtained by using the two focusing algorithms described above, namely, the conventional DAS algorithm and the correlation method with highest potential (TCC back projection). However, before these algorithms can be applied, the tumor response must be extracted from the data sets. Unwanted signals were subtracted as discussed above by RLS-filtering. The efficacy of the artifact-removal RLS-algorithm is demonstrated in Figure 6 for a representative subset of received waveforms. The overall concordance between full wave simulations and experiment is good but, as expected, with more clutter in the late-time response of measured data. The expected arrival times obtained from simulations also fit well with those measured indicating a nearly constant phase center for these viewing angles. 

**Figure 7:**
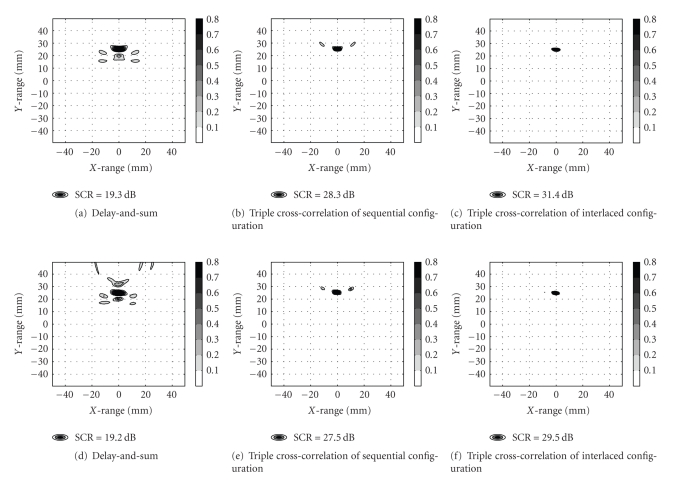
Grey-scale images of power deposition in numerical (upper panels) and experimental (lower panels) runs using 16 antennas.

In Figure 7, 2D images of the scattered energy versus position are presented for 16 antennas as contoured maps on a linear scale. The strongest scatterer is in all cases located at the correct position P_*T*_(*x* = 0.0 mm, *y* = 25.0 mm). However, the SCR differs significantly between the imaging algorithms. First, TCC beamforming outperformed DAS beamforming with 8.3 dB (sequential configuration) and 10.3 dB (interlaced configuration) smaller SCR values, respectively, using experimental data. Similar numbers were obtained (9 dB and 12.1 dB) for the numerical runs. 

Second, from Figure 7 it is evident that spatial resolution is also particularly improved when comparing DAS and TCC beamforming.  Table 2 quantifies the performance indices (SCR and FWHM) versus number of antennas. As intuitively expected, the DAS algorithm improves with increasing number of antennas through better SCRs and smaller FWHM-values. Furthermore, the TCC algorithm outperformed the DAS algorithm for all cases with interlaced configuration being superior to sequential configuration. Comparing TCC sequential configuration with DAS, typically >10 dB improved SCR and ~50% smaller FWHM are observed for the former method. Surprisingly, improved resolution is also obtained along the axial (longitudinal) direction which may be attributed to scanning of the target from all viewing angles. To support the experimental study, full wave numerical runs were undertaken to verify the findings. The concordance in estimated values of the performance indices was good as can be seen by comparing Tables 2 and 3. Overall, SCR for the numerical runs were a few tenths of a dB smaller compared to values obtained from experimental data. The same applies to the FWHM-parameter where typical deviations between numerically and experimentally generated values were about 0.2 mm. 

## 5. Discussion

Imaging based on electromagnetic waves has previously been implemented in applications such as nondestructive testing (NDT), ground penetrating radar (GPR), through-wall radar, medicine asf. Advances in circuit technology and unlicensed use of the frequency range from 3.1 to 10.6 GHz (authorized by the Federal Communications Commission (http://www.fcc.gov/)) have initiated innovations related to short-range radar UWB applications. Common to all techniques is the need for a robust imaging algorithm that solves the inverse problem and produces informative images of the scene under investigation. We have selected the widely used delay-and-sum (DAS) beamforming algorithm as a benchmark for this study. The DAS scheme is a simplified variant of the so called Microwave Imaging via Space Time (MIST) beamforming technique suggested by Bond et al. [18]. The latter approach also contains, apart from the time alignment of signals, a finite impulse response (FIR) filter that removes path length-dependent dispersion and attenuation of the various channel responses. However, since the present phantom model adds little distortion (negligible dispersion and attenuation) to the propagating pulses, the above FIR-filter has been neglected in the analysis. Nevertheless, we emphasize that the MIST algorithm can be generalized by implementing cross-correlation back projection (presently under study) instead of DAS beamforming.

The aim of in this paper was to evaluate a new method for UWB imaging based on the cross-correlated back projection scheme [15]. Similar to the widely used delay-and-sum algorithm, the cross-correlated back projection approach is also based on estimated round trip time-of-flights from a stimulating source to a focal point and back to a receiver. Furthermore, the spatial position of an assumed object is found as an intersection of several baselines (ellipses) related to the time-of-flights. Using the same test scenarios, ranging from analytical to numerical and experimental setups, we have evaluated the performance of cross-correlated back projection against conventional DAS beamforming.

According to (3), the DCC algorithm requires a reference receiver to perform correlation. This raises the question regarding optimum relative localization of antenna *A*_*i*_ versus *A*_*i*_^*R*^. Two basic criteria are to be respected in order to maximize performance. First, maximum orthogonality between the receiver signals should be aimed at. This is equivalent to a large relative antenna distance *D*, as lateral resolution is degraded when the antennas are located closer to each other (see (4)). A second criterion is similarity in pulse shape (same antenna point response) to yield a large correlation coefficient at the focus point. As the scattering response of real objects is strongly dependent on incidence/scattering angle, and the cross-correlation algorithm assumes that the point responses are identical, the distance between *A*_*i*_ and *A*_*i*_^*R*^ must be kept small relative to the paired antenna-to-object distances. Hence, dependent on the observation scenario and considering the above tradeoff, an intermediate distance (that satisfies both requirements) should be selected when pairing *A*_*i*_ and *A*_*i*_^*R*^. Our findings confirmed this property since interlaced configuration (nearly the same observation angle, but still provides some distance between receivers) was superior to sequential combination. 

 Other general aspects of imaging algorithm efficiency are robustness, execution time, and computational complexity. No additional * a priori* information or assumptions regarding the setup are required for the cross-correlation scheme when compared to the DAS-algorithm. However, in order to localize a scattering object correctly in space, the transmission medium velocity is assumed to be known and constant. The robustness of cross-correlated back projection applied to heterogeneous media is a critical factor that needs to be addressed in future work. As for algorithm execution time, using vectorized implementation for signal products to avoid program loops, the TCC-algorithm was observed to be only marginally slower than the DAS-algorithm. In addition, the low computational complexity of the DAS-algorithm is preserved in the correlation approach. 

## 6. Conclusion

We have presented an experimental characterization, supplemented by numerical modeling, of a monostatic radar-based system for breast cancer detection. The experimental setup is quasi 2D with tissue mimicking materials that give adequate values of dielectric contrast both between normal breast tissue and malignant tissue as well as normal breast tissue and skin. Our findings confirm the superior performance of this novel cross-correlation scheme in terms of reduced clutter (sidelobes) and improved spatial resolution in focused 2D images. A systematic study, where the number of antennas was varied, showed that typically the SCR is increased with about 10 dB. In addition, the spatial resolution (full-width half maximum of the imaged peak) is at least 50% better along the lateral direction and typically 40% along the axial direction. To the best of our knowledge, experimental results from using cross-correlated back projection in UWB imaging, have not been presented in the open literature before.

## Figures and Tables

**Table 1:** FWHM and SCR for different imaging methods in numerical test scenario.

Method	SCR (dB)	FWHM_*x*_ (mm)	FWHM_*y*_ (mm)
Delay-and-sum	19.1	6.3	2.8
Dual cross-correlation	28.0	2.9	2.6
Triple cross-correlation	30.0	2.9	2.5

**Table 2:** SCR and FWHM from experiments with different number of antennas.

# of ant	Delay-and-sum			Triple cross-correlation					
				Sequential			Interlaced		
	SCR (dB)	FWHM_*x*_ (mm)	FWHM_*y*_ (mm)	SCR (dB)	FWHM_*y*_ (mm)	FWHM_*y*_ (mm)	SCR (dB)	FWHM_*y*_ (mm)	FWHM_*y*_ (mm)
4	14.4	10.8	3.6	26.0	4.1	1.5	—	—	—
8	17.3	7.1	2.8	28.5	3.8	1.9	32.0	3.9	1.3
16	19.2	7.4	2.9	27.5	4.0	2.3	29.5	3.5	1.7
24	22.0	6.9	2.7	25.3	4.4	2.4	32.2	3.4	1.7

**Table 3:** Same as Table 2 for numerical computations.

# of ant	Delay-and-sum			Triple cross-correlation					
				Sequential			Interlaced		
	SCR (dB)	FWHM_*x*_ (mm)	FWHM_*y*_ (mm)	SCR (dB)	FWHM_*y*_ (mm)	FWHM_*y*_ (mm)	SCR (dB)	FWHM_*y*_ (mm)	FWHM_*y*_ (mm)
4	14.6	10.2	3.8	27.1	4.2	1.6	—	—	—
8	17.6	7.0	3.0	29.3	3.8	1.7	32.2	3.9	1.5
16	19.3	6.9	2.9	28.3	3.8	2.0	31.4	3.7	1.6
24	21.9	6.8	2.9	26.0	4.3	2.3	32.4	3.7	1.5
